# The perceived beauty of art is not strongly calibrated to the statistical regularities of real-world scenes

**DOI:** 10.1038/s41598-024-69689-6

**Published:** 2024-08-21

**Authors:** Alexander Swartz, Alice E. Skelton, George Mather, Jenny M. Bosten, John Maule, Anna Franklin

**Affiliations:** 1 The Sussex Colour Group, The School of Psychology, University of Sussex, Brighton, BN1 9RH UK; 2 Nature and Development Lab, The School of Psychology, University of Sussex, Brighton, BN1 9RH UK; 3 The School of Psychology, University of Sussex, Brighton, BN1 9RH UK; 4 Sussex Vision Lab, The School of Psychology, University of Sussex, Brighton, BN1 9RH UK; 5 Statistical Perception Lab, The School of Psychology, University of Sussex, Brighton, BN1 9RH UK

**Keywords:** Human behaviour, Psychology

## Abstract

Aesthetic judgements are partly predicted by image statistics, although the extent to which they are calibrated to the statistics of real-world scenes and the ‘visual diet’ of daily life is unclear. Here, we investigated the extent to which the beauty ratings of Western oil paintings from the JenAesthetics dataset can be accounted for by real-world scene statistics. We computed spatial and chromatic image statistics for the paintings and a set of real-world scenes captured by a head-mounted camera as participants went about daily lives. Partial least squares regression (PLSR) indicated that 6–15% of the variance in beauty ratings of the art can be accounted for by the art’s image statistics. The luminance contrast of paintings made an important contribution to the PLSR models: paintings were perceived as more beautiful the greater the variation in luminance. PLSR models which expressed the art’s image statistics relative to real-world scene statistics explained a similar amount of variance to models using the art’s image statistics. The importance of an image statistic to perceived beauty was not related to how closely art reproduces the value from the real world. The findings suggest that beauty judgements of art are not strongly calibrated to the scene statistics of the real world.

## Introduction

Why some photographs, artworks or landscapes are aesthetically preferred is a question that continues to challenge cognitive science. The foundation for investigating the relationship between aesthetic experiences and measurable properties of visual stimuli was laid with the nineteenth century work of Gustav Fechner^1^. Since then, computational aesthetics has achieved some success at modelling aesthetic preferences using image properties^2^, and visual properties such as edge density, luminance, and saturation contrast have been found to be related to aesthetic appreciation of scenes, faces, objects and art^3–6^.

Related to the notion that aesthetics can be partially accounted for by visual properties, vision scientists have also suggested that there is a relationship between aesthetics and the statistical regularities of real-world scenes, known as ‘natural scene statistics’^7–9^. One natural scene statistic, fractal dimension, is a measure of the extent to which self-repeating patterns across scales fill a space^10^. Natural scenes generally have a mid-range fractal dimension^11^, and aesthetic ratings of computer-generated fractal patterns suggest that, on average, patterns with a mid-range fractal dimension are most aesthetically pleasing (e.g.,^10^). Another natural scene statistic is that of spectral slope which describes the Fourier amplitude of spatial information over different spatial scales, with natural scenes having a characteristic spectral slope function of $1/{f}^{\alpha }$ where f is spatial frequency and *α* is approximately 1.00–1.25 (e.g.,^12^). Judgements of artistic merit have been found to peak, and visual discomfort has been found to be lowest, for abstract noise patterns that have the spectral slope typical of natural scenes^13^. In the color domain, the chromaticities of natural scenes tend to form a distribution that stretches along the blue-yellow color appearance dimension (the negative diagonal axis in the cone-opponent MacLeod-Boynton^14^ chromaticity diagram)^15,16^. As with spectral slope, it has also been shown that artistic merit peaks, and visual discomfort is lowest, for colored ‘Mondrians’ approximately of 1/f spectral slope, composed of overlapping colored rectangles which conform to the blue-yellow chromatic distribution of natural scenes^13^.

These converging lines of evidence that aesthetic judgments such as artistic merit are related to natural scene statistics could suggest that the sensory component of aesthetic experience^17^ is broadly calibrated to natural scenes^8,9^. This idea links to the theory of biophilia, which suggests that humans like nature, and that it benefits them, because humans have evolved and are adapted to operate in natural environments^18^. The idea that aesthetics is calibrated to natural scene statistics also relates to the efficient coding hypothesis which proposes that human visual systems have evolved to efficiently represent natural scenes^9,19^. Efficient coding could mean that images with natural scene statistics are more fluently processed and therefore are aesthetically preferred to images with visual properties that are not typical of natural scenes^20,21^. Another potential mechanism, that is possibly related to efficient coding, is that adaptation to the real-world scenes typically encountered in daily life could reduce sensory sensitivity to the statistical regularities of those scenes, making images with those scene statistics more comfortable to view and thus more liked^13^. Note that this proposal could apply to many different types of real-world scene and not just scenes of nature.

As outlined above, vision science has pointed to a relationship between aesthetic judgements and natural scene statistics, with preferences peaking for abstract patterns with statistical regularities typical of real-world scenes^10^. This proposal has led to consideration of whether natural scene statistics more broadly have a role in the aesthetics of art. Several studies have shown that art often has spatial properties that mimic the statistics of natural scenes, even for abstract art^21–23^. Jackson Pollock’s abstract drip paintings, for instance, have a fractal dimension similar to that of natural scenes^24^. There is also evidence that spatial natural scene statistics predict aesthetic experience for some types of art. For example, departure from the 1/f spectral slope typical of real-world scenes also predicts visual discomfort for non-representational art^25,26^. However, other studies which have modelled aesthetic judgements of art with a broader range of image statistics potentially question the role of natural scenes. For example, one study used the JenAesthetics dataset^27–29^ to analyze the contribution of a set of image statistics to ratings of artistic value and beauty for 1614 Western oil paintings from 11 major art periods^30^. Image statistics such as self-similarity and anisotropy predicted artistic value and beauty, although which image statistics were significant also varied across different art periods. These findings potentially suggest that the aesthetics of art may be too variable to be universally accounted for by one set of statistical properties characteristic of natural scenes. Mather^5^ further analyzed the beauty ratings of the JenAesthetics data set as well as the MART database of emotion ratings for a set of abstract art^31^ and analyzed the contributions of spectral slope, fractal dimension and entropy for chromatic and luminance channels. Mather found that these image statistics predicted aesthetic judgements, but he also found that the predictive image statistics varied across genres of art. For example, steeper spectral slopes were preferred for portraits whereas shallower spectral slopes were preferred for nude artworks. This variation across genres again questions the notion that aesthetic judgements of art are broadly calibrated to the statistical properties of natural scenes.

For the case of color, the blue–yellow variation characteristic of natural scenes is also present in some types of artworks, albeit slightly biased towards red hues^32–34^. Multiple studies have also shown that people also tend to prefer art, even abstract art, in its original color composition (e.g.,^35–38^), and people also seem to prefer images with chromatic distributions that appear natural^39^. A recent study by Nakauchi and Tamura^40^ explored whether the color statistics that predict aesthetic ratings of art are similar to those of natural scenes. A set of 1200 paintings from WikiArt were shown in their original color composition alongside three versions where the painting’s hues had been manipulated to varying degrees, and 31,353 participants were asked to select their preferred painting from each set of four in an online task. The contributions of the mean, variance and skew of the dimensions of the CIE L*a*b* perceptual color space^41^ and the correlations between these dimensions and preference judgments were evaluated with multiple regressions. Nakauchi and Tamura^40^ also analyzed the color statistics of a set of 1200 outdoor natural scenes from the SUN database. Certain color statistics such as the skewness of a* (a* quantifies the redness-greenness of a color) predicted preferences for the paintings. However, there were also significant differences between the color statistics of the paintings and those of the outdoor natural scenes, and the preferred color composition of paintings was not that which was typical of natural scenes. Whilst these findings appear to suggest that preferences for paintings are not calibrated to the color statistics of natural scenes, the extent to which the color statistics characteristic of the outdoor scenes predict preferences for the paintings was not directly quantified.

The current study further investigates the relationship between aesthetic judgements of art and natural scene statistics. The study specifically aims to directly quantify the extent to which statistical regularities of real-world scenes predict beauty judgements of art. As in Refs.^5,30^, we analyze the beauty ratings of the JenAesthetics dataset rather than other measures such as artistic merit. We chose to analyze perceived beauty as we felt that perceived beauty is a clearer and more intuitive concept for participants to rate, and is also easier to interpret than artistic merit. We make the data and scripts of the current study available for those who wish to apply our approach to the other measures of the JenAesthetics dataset. We selected a subset of 785 oil paintings for analysis including artworks across multiple genres as well as two further subsets of 276 landscape artworks and 519 portrait artworks. Like Mather^5^ we analyze fractal dimension, spectral slope and entropy of the images of the paintings (although only for the luminance channel), and we also analyze the lacunarity (heterogeneity of spatial patterns comprising an image)^42^; and edge density^43^ as these are also important spatial image statistics predictive of aesthetics^6^. We also include a set of chromatic statistics defined in a biologically plausible color space^14^, which enables us to define colors in terms of their activation of the two cardinal neural subsystems underpinning color vision. Much prior work on aesthetics (e.g.^4,30^) uses color spaces (e.g., HSV) that are suitable for computer graphics but that do not accurately represent human color vision (linear analyses of hue are also problematic as it is a circular quantity). Perceptual color spaces (e.g., CIELAB and CIELUV, as in refs.^5,40^.) are more appropriate than HSV. We use the MacLeod-Boynton^14^ chromaticity diagram, as this enables us to understand the contributions of the biological components of color vision to aesthetic judgements of art, as well as the contributions of the dimensions of saturation and luminance. We compute the standard deviation of saturation and luminance as these have proved important in predicting the pleasantness of van Gogh’s landscapes^6^. We also include two chromatic statistics which quantify the elongation and angle of the chromatic distribution, and which provide an efficient way of characterizing the extent to which the chromatic distribution is blue-yellow biased as in natural scenes^16,44^. We use partial least squares regressions (PLSRs) to investigate whether the chromatic and spatial image statistics of the paintings predict participants’ beauty judgements of the art. We also conduct permutation analyses on random iterations of the preference data to confirm that the variance explained by the PLSRs is significant.

Given the findings in prior studies that predictive image statistics vary for different types of art, we analyze across all categories of the JenAesthetics dataset but also conduct separate analyses for landscapes and portraits, as these categories both had sufficient numbers of images for separate analysis. Different relationships between beauty judgements and real-world scene statistics might be expected for the two genres since they differ in their spatial scales, and may also differ in the extent to which they recruit higher-order processes for judgements of aesthetics.

Importantly, we directly test the hypothesis that aesthetic judgements of art are calibrated to natural scene statistics by analyzing the extent to which the chromatic and spatial image statistics predict the beauty ratings of the paintings when expressed relative to the values of image statistics for a set of real-world scenes. We analyze a set of egocentric real-world scenes captured by head-mounted cameras^45,46^ to provide the normative data. The image set captures real-world scenes as people go about their daily lives, in different kinds of real-world environments both urban and rural, indoors and outdoors. Whilst Nakauchi and Tamura^40^ considered the statistics of photographs of outdoor nature scenes, if aesthetic judgements are calibrated to natural scene statistics through adaptation to the statistics of scenes that people are immersed in, then the distinction between indoor and outdoor scene types should not be important—what would be important would be the scene statistics of the observer’s ‘visual diet’. The image set aimed to capture scenes that approximate the viewpoints of people when going about daily lives, rather than composed photographs of nature. We therefore consider the head-mounted camera real-world scene set that we use to be more ecologically valid than other natural scene image sets for addressing the question of the extent to which beauty calibrates to the real-world scene statistics of one’s visual diet. We compute the chromatic and spatial image statistics of the paintings and express these relative to the mean and standard deviation of the real-world image data set, providing a measure of similarity to the real-world scenes (smaller z-scores indicate greater similarity to real-world scenes). We then repeat the PLSRs on the beauty ratings using the scene statistics expressed relative to those of real-world scenes to quantify the extent to which similarity to real-world scene statistics predicts the perceived beauty of art. If the beauty of art is calibrated to real-world scenes, we expect that the PLSRs will explain more variance in beauty judgements when the art's image statistics are expressed relative to the distribution of image statistics of real-world scenes.

## Results

**Figure 1:**
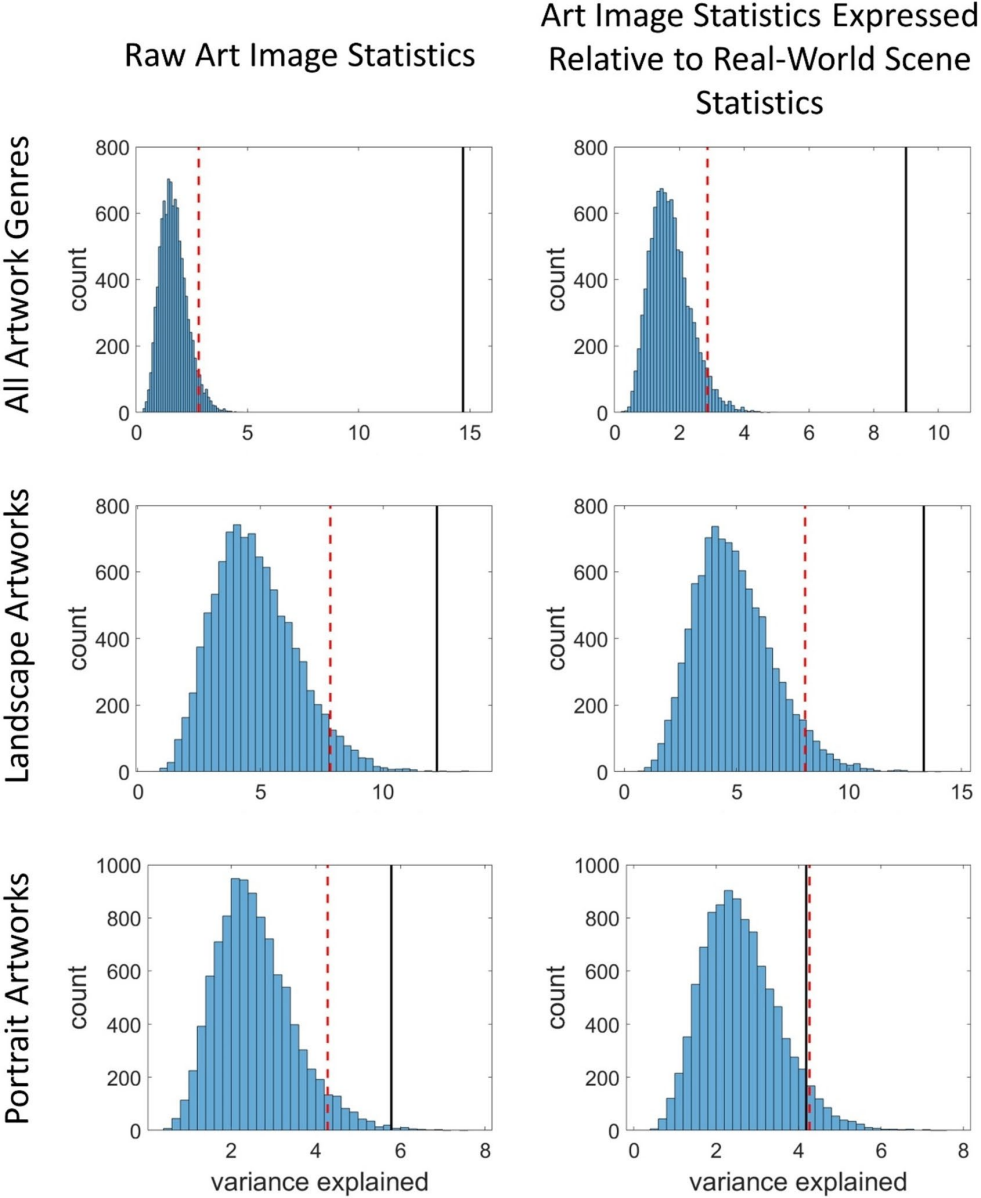
Histograms show the variance explained by a set of 10,000 permuted PLSRs with random permutations of the beauty ratings, with the 95th percentile of variance explained in the set of permuted PLSRs indicated with the red dashed line. The variance explained by the non-permuted PLSR is indicated with a solid black line. Variance explained is given for PLSRs using the art’s raw image statistics (left hand column) and the art’s image statistics expressed relative to the real-world (right hand column), for all genres of paintings (top row), landscapes (middle row) and portraits (bottom row).

The PLSR analysis on the raw image statistics of all paintings (i.e., without expressing relative to the statistics of images from the real world) found that chromatic and spatial image statistics of the art predicted 14.69% of the variance in the JenAesthetics beauty ratings (with 3 components). The PLSR on landscapes alone accounted for 12.20% of the variance (with 2 components), while the PLSR on portraits alone accounted for 5.79% of the variance (with 3 components). The standard deviation of luminance was an important contributor to all three models as indicated by high ‘variable importance in projection’ (VIP) scores (see Methods for definition and Supplementary Table S1 for values of VIP scores). The variance explained in these PLSRs was greater than the 95^th^ percentile of variance explained in a set of 10,000 permuted PLSRs with random permutations of the beauty ratings: all genres (2.80%), landscapes (7.85%) and portrait images (4.28%, see Fig. 1). A further set of PLSRs using image statistics expressed relative to typical values from the real world found that the similarity of the art’s image statistics to real-world scenes explained 9.00% of the variance in the beauty ratings of all genres of art (3 component model), 13.24% for landscapes (2 component model) and 4.18% for portraits (2 component model). The variance explained by these PLSRs was greater than the 95th percentile of variance explained in a set of 10,000 permuted PLSRs with random permutations of the beauty ratings for all genres (2.87%) and landscapes (8.05%) but not for portraits (4.27%, see Fig. 1).

Table S1 gives the PLSR VIP scores which identify the contributions of the art’s raw image statistics and the art’s image statistics expressed relative to real-world values, to beauty ratings of art for all-genre and landscape analyses. As the real-world model of the beauty ratings of portraits did not explain more variance than the 95th percentile of the variance explained by the model under the null hypothesis using permuted data, we do not interpret the VIP scores of the PLSR for portraits. As the table shows, some image statistics make an important contribution to the model (VIP > 1.25). For the PLSRs that use the art’s raw image statistics, standard deviation of luminance makes an important contribution to the models for all genres, for portraits and for landscapes. For landscapes, curved edge density additionally makes and important contribution, and for portraits spectral slope and straight edge density also make important contributions. For PLSRs that use image statistics expressed relative to the real world, the mean S/(L + M), angle of maximum color variance and spectral slope make important contributions for all genres, and straight and curved edge density and standard deviation of S/(L + M) make important contributions for landscapes. Figure S1 gives further information to aid the interpretation of the VIP scores for the image statistics by showing the distributions of the art’s raw image statistics and the mean statistics from the real-world images used to calculate the art’s image statistics expressed relative to real-world values.

Because the mean image statistic from the real-world scenes was often not centrally located in the distribution of the art image statistic (Fig. S1), the image statistics expressed relative to real-world values were not independent of the raw image statistics. It is possible that the better than chance performance for image statistics expressed relative to the real world (Fig. 1) could be accounted for by the fact that these statistics are correlated with the raw statistics, if the latter themselves explain variance in ratings of beauty (left column of Fig. 1). We therefore conducted two additional analyses to further test the hypothesis that perceived beauty is calibrated to real-world image statistics and to investigate whether the two sets of predictors (raw image statistics vs. image statistics relative to real-world values) perform differently from one another. First, we correlated the VIP scores from the PLSR on the raw image statistics with the corresponding absolute differences between the mean real-world image statistics and the mean art statistic, expressed as a z-score. If perceived beauty is calibrated to real-world scene statistics, then the art image statistics which are most similar to the real-world values should have the higher VIP scores in the PLSRs (in other words, there should be a negative correlation between VIP score and z-score). There was no significant correlation between the size of the differences between the real-world scene and art means (expressed as z-scores) and VIP scores across the image statistics for all genres (rho = − 0.12, p = 0.68) or for landscapes only (rho = − 0.24, p = 0.39), although both were negative as predicted. Therefore, we did not find that the VIP scores that contribute more to the models were for image statistics where the values for art are closer to those of the real world.

**Figure 2:**
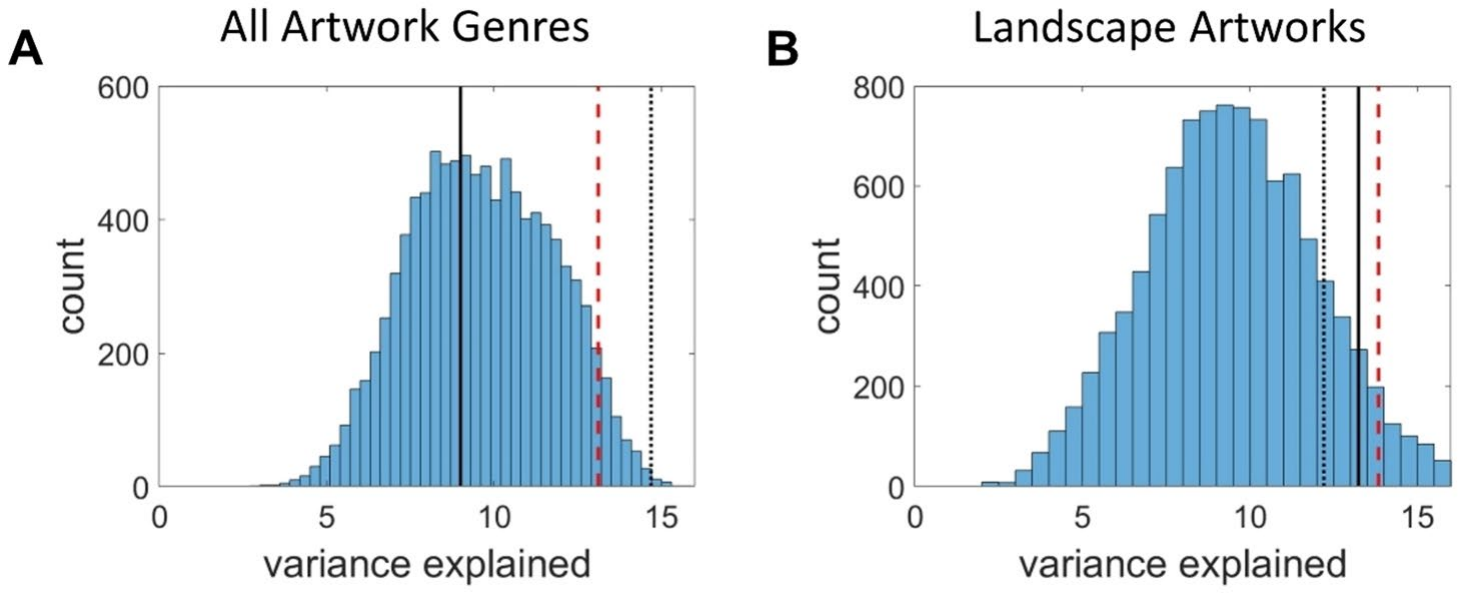
Distributions of variance explained with 10,000 iterations of the PLSR analysis where the art’s image statistics are each expressed relative to random values selected from the art’s distribution of that image statistic, rather than relative to the real-world values. The dashed red line gives the 95th percentile of the variance explained by the 10,000 iterated PLSRs that express the image statistics relative to randomly selected values. The dotted black line gives the variance explained by the PLSR using the art’s raw image statistics and the solid black line gives the variance explained by the PLSR using image statistics expressed relative to real-world values. Panel A shows results for all artwork genres and panel B shows results for landscape artworks only.

Second, we compared the variance explained in perceived beauty by the art's image statistics and by the image statistics expressed relative to the real world, with the variance explained by the image statistics expressed relative to arbitrary reference values. If perceived beauty is strongly calibrated to the real world, then the variance explained by the real-world PLSR should outperform the majority of models calibrated to arbitrary values. We expressed image statistics relative to arbitrary reference values from the art’s image statistic distributions rather than the average value from the real-world scenes. We conducted 10,000 iterative PLSRs each time with different randomly selected arbitrary reference values for each image statistic. Figure 2 gives the distribution of variance explained by the 10,000 iterations of the PLSR conducted using the art's image statistics expressed relative to randomly selected arbitrary reference values from the art's image statistic distribution. As can be seen from the figure, for both all genres and for landscapes, the percentage of variance explained by art’s image statistics when expressed relative to real-world scenes is not greater than the 95th percentile of variance from the iterated PLSRs which express the art’s image statistics relative to arbitrary values randomly selected from the art’s distributions of image statistic values. In other words, expressing art image statistics relative to real-world scenes does not explain more variance than expressing art image statistics relative to randomly selected values. For the analysis of all genres of art, the image statistics expressed relative to the real world explain about the average amount of variance explained by arbitrary values, whereas the raw image statistics explain more variance than the 95th percentile of PLSR iterations. For the landscapes, both the raw image statistics and the image statistics expressed relative to the real world explain about the same amount of variance as the 95th percentile of PLSR iterations for the arbitrary values.

## Discussion

We aimed to further understand the role of scene statistics in aesthetics and to establish the extent to which the perceived beauty of art is calibrated to the statistical regularities of real-world scenes. We found that a set of spatial and chromatic image statistics could predict about 6–15% of the variance in ratings of the beauty of a large set of Western oil paintings spanning 11 art periods. The amount of luminance contrast in the paintings strongly contributed to the models when all genres of art were analyzed as well as for landscapes and portraits separately—suggesting that people find Western oil paintings more beautiful the greater the variation in luminance. When the image statistics were expressed relative to the statistics of a set of real-world scenes captured with calibrated head-mounted cameras, the relative scene statistics predicted a similar amount of variance in the ratings of beauty of the art (4–13%) to that predicted by the raw image statistics. The variance in perceived beauty ratings accounted for by relative image statistics was somewhat less than that of the art’s raw image statistics for the all-genre analysis and for portraits, but was slightly more for landscapes. Image statistics expressed relative to typical values for real-world images also predicted the beauty of art better than permuted data for the whole set of art and for landscapes, but not for portraits. However, further analysis showed that the importance of an image statistic to the model of perceived beauty was not related to how closely art reproduces the value from the real world. Additionally, a model based on image statistics expressed relative to the average statistics of the real-world scenes did not outperform models based on image statistics expressed relative to randomly selected arbitrary values from the distributions of the art’s image statistics: the variance explained was not greater than the 95th percentile of the variance explained by PLSRs using the arbitrary image statistic values.

For PLSRs based on raw image statistics, standard deviation of luminance was a strong contributor to the model for all genres of art, and for portraits and landscapes separately (see also^6^). For PLSRs based on image statistics expressed relative to real-world values, six statistics (curved edge density, straight edge density, the mean and standard deviation of S/(L + M), spectral slope and the angle of the maximum color variance) made important contributions to the model (VIP > 1.25). However, this can not necessarily be interpreted as in support of the hypothesis that perceived beauty is calibrated to real-world values for these image statistics: for four of these statistics, the mean real-world statistic lay at one extreme of the distribution of art statistics so that the statistics when expressed relative to the real-world statistic only had minor differences from the original raw statistics. Such systematic differences between mean statistics of art and mean statistics of real-world images could themselves be interpreted as evidence against a ‘real-world calibration’ hypothesis. To the extent that art is intended to be aesthetically pleasing, the distributions of art statistics might be expected to be close to the real-world means if aesthetic judgements occur for image statistics well matched to real-world scenes.

There has been much research attempting to model aesthetic judgements using a wide range of visual properties and image statistics^3–6,30,47^. The amount of variance explained here with our set of spatial and chromatic image statistics is similar to that of other studies (e.g., 8% in Ref.^48^ for a set of 15 image features, see also Refs.^4,6^). The main contribution of the current study is that we targeted analyses to assess the extent to which beauty is calibrated to the spatial and chromatic image statistics of real-world scenes. Our finding that the image statistics when expressed relative to typical values of real-world scenes accounts for a similar amount of variance in beauty ratings to raw values of those image statistics, challenges the hypothesis that types of aesthetic judgements are strongly calibrated to the statistical regularities of real-world scenes^8,21^. We make no claim here that our finding applies to all types of aesthetic judgement. Here, we chose to analyze the beauty ratings of the JenAesthetics dataset as we felt that beauty was a more intuitive concept than the dataset’s aesthetic quality measure. Although both measures are correlated (*rho* = 0.78), subtle differences in the relationships of these measures with real-world scene statistics may well be found. Relationships with other measures of the JenAesthetics dataset could also be analyzed, such as the extent to which image color preference is calibrated to real-world chromatic statistics, and we make our image analysis and analysis scripts available to facilitate this.

Prior studies have found that abstract patterns with a spectral slope or fractal dimension typical of natural scenes are preferred^10^. Here, spectral slope expressed relative to the real-world statistics makes an important contribution to the all-genre and landscape models (see VIP scores Table S1). However, further inspection of this result reveals a positive relationship between spectral slope expressed relative to the real world and perceived beauty, suggesting that, in fact, perceived beauty increases the greater the deviation of the art’s spectral slope from real-world scenes. As shown in Fig. S1, for spectral slope, the values for the real-world scenes tended to be lower than those of the art’s image statistics, and so one interpretation of the increase in beauty with greater deviation in the image statistics from real-world scenes is that art judged as beautiful accentuates some of the visual properties typical of real-world scenes^49^. Further research using the approach of the current study, but where the range of art includes works with image statistics spanning both sides of the real-world distributions more evenly, could help determine whether or not this interpretation is valid.

Consistent with prior research, we also found that the contribution of image statistics to a type of aesthetic judgement (in this case perceived beauty) varies with the genre of art^5^. Most notably, image statistics expressed relative to those of the real world predict more variance in beauty judgements than the art’s raw image statistics, and more variance than the permuted image statistics under the null hypothesis, for landscapes but not for portraits. One possibility is that the real-world scenes of daily life, as captured by a head mounted camera, are more relevant to the perceived beauty of landscapes than for other art genres. The perceived beauty of other art genres such as portraits might also be more strongly influenced by higher-order processes than that of landscapes. Further research which analyzes other art databases for a broader range of art and which considers other art genres separately is needed to clarify the conditions under which real-world scene statistics are predictive of perceived beauty.

To address the hypothesis that perceived beauty is calibrated to real-world scenes, we chose to extract ‘real-world’ statistics from images randomly sampled using calibrated head-mounted cameras. We chose to define ‘real-world scenes’ as images of the daily visual environments that participants experience, as this is compatible with ideas of visual calibration via adaptation^50,51^, where the visual system adapts to efficiently represent to the range of image statistics it encounters or, alternatively, via ‘self-referential’ processing^48,52^, where people may make aesthetic judgements with reference to their own identities and experiences. A different strategy would have been to extract the statistics of a set of images from ‘natural’ environments, defined as the ancestral environments in which humans evolved. Any modern dataset could, of course, only approximately meet this aim. Further work including cross-environmental studies on aesthetics which investigate whether environmental differences in visual diet (e.g., across locations or seasons) correspond with differences in aesthetic judgements, and which examine whether calibration is stronger to more naturalistic environments, would be valuable to further understand whether any aspects of aesthetics are calibrated to real-world scenes within a lifetime or through evolution^45^.

In sum, we found that perceived beauty is not strongly calibrated to the statistics of real-world scenes. Statistics expressed relative to typical statistics of real-world images account for a somewhat smaller percentage of variance in judgements of the perceived beauty of art across all genres than statistics expressed in their original scales. For landscapes, beauty perception may be better related to real-world scene statistics: here, statistics expressed relative to real-world values accounted for slightly more variance in beauty judgements than statistics expressed in their original forms. However, the importance of an image statistic to the model of perceived beauty was not significantly related to how closely art reproduces the value from the real-world. Rather than the findings pointing to a strong calibration of perceived beauty to real-world scenes, the results suggest that people find Western oil paintings more beautiful the greater the painting’s variation in luminance. The relatively low variance in the ratings explained by image statistics overall suggests that other factors, such as meaning and semantic content, play a crucial role in aesthetic judgements of beauty.

## Methods

### JenAesthetics art images and beauty ratings

**Figure 3:**
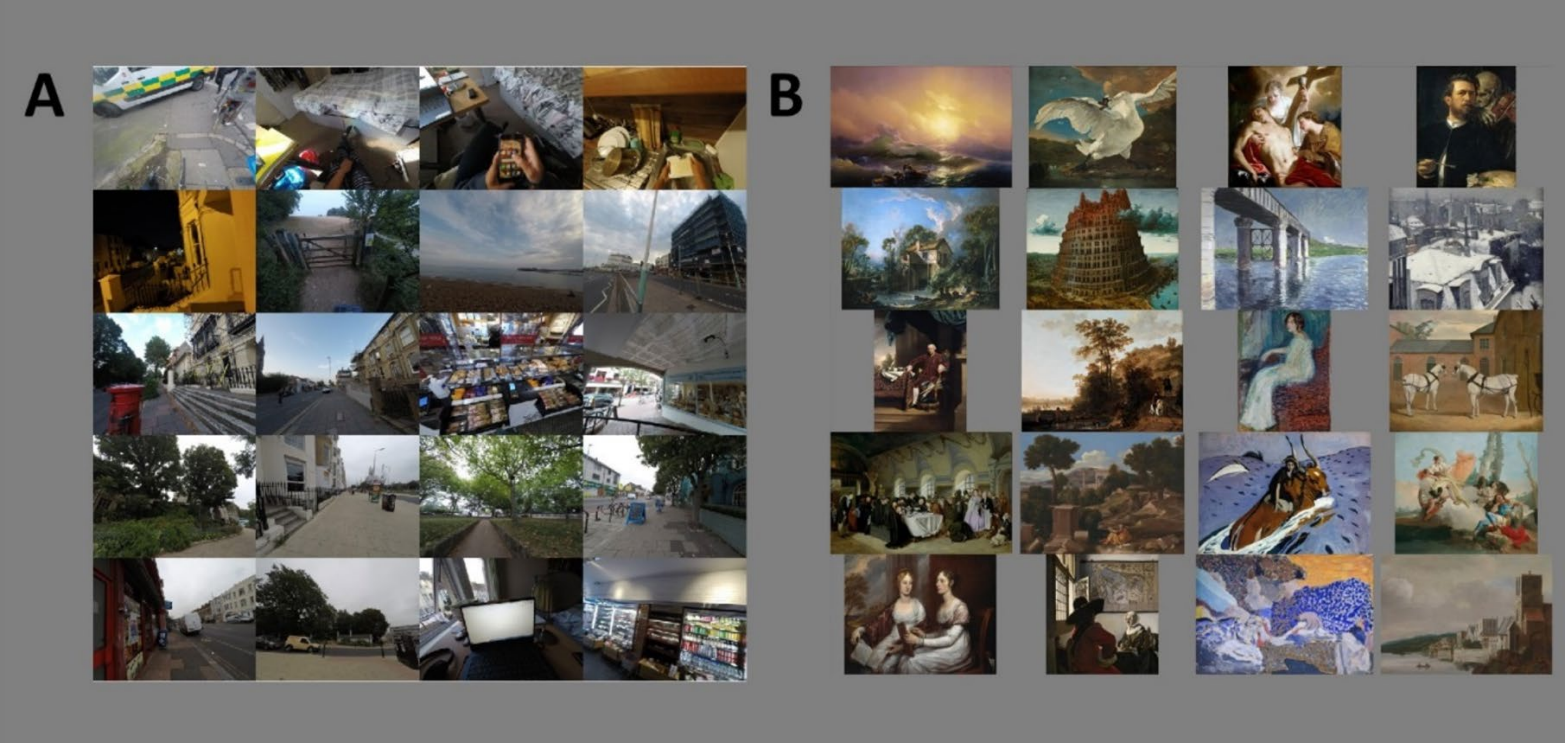
Montages of a random selection of the images used in the study. (**A**) A selection of images from the real-world scenes set^45,46^. (**B**) A selection of the public domain subset of art images from the JenAesthetics database^27–29^.

We used the art images and subjective aesthetic ratings from the JenAesthetics dataset^27–29^, which includes images of artworks by various artists, from various time periods and from various genres of art, and includes 16 different subject matters including abstract, landscape, still life, portraits, nude, urban scene, animal and scenes with people (see Fig. 3). Of the 1628 total images of paintings from 410 artists, we identified a subset of 785 images for our analysis. Our selection process for this subset of the images sought to address overrepresentation of any artist by randomly selecting 3 images for a given artist where more than 3 artworks were included in the dataset for that artist (following Mather^5^). The JenAesthetics dataset includes several different types of subjective ratings gathered from 134 mostly student participants (19–24 years old, all studying fields unrelated to art and all providing written informed consent to participate) living in the city of Jena in central Germany^27^. Each participant rated a unique subset of 163 images, and across the dataset each image was rated by 19–21 different individuals. Here we analyze the ‘beauty’ ratings of the JenAesthetics artworks, where participants were asked ‘how beautiful is the image?’ on a 1–100 scale with ‘not beautiful’ on the left side of the scale and ‘beautiful’ on the right, in order to operationalize a measure of aesthetic preference. We analyzed the beauty ratings, rather than the JenAesthetics aesthetic quality measure, as we felt beauty is a more understandable and clearer concept to rate and interpret. We analyzed the relationship between real-world scene statistics and perceived beauty for the full set of selected art images which included the following ‘genres’ of art: abstracts, landscapes, people, still lifes, portraits, nudes, animals and built environments. We also examined this relationship for two further subsets of all of the landscape artworks (n = 276) and all of the portrait artworks (n = 519) included in the JenAesthetics set, as categorized in Ref.^5^. For these subset analyses, we did not constrain the number of works that each artist contributed to the landscape and portrait image sets to ensure that there were sufficient numbers of art images and ratings to analyze.

### Real-world scene set

Our real-world scene set consisted of images collected in and around the city of Brighton and Hove in Southern England and were gathered as part of a project which aimed to capture the chromatic scene statistics and ‘visual diets’ of daily life in different environments^45,46^. The images were collected by 8 participants (5 female, 3 male) with a mean age of 34.7 (*SD* = 4.0) who wore color-calibrated head-mounted GoPro cameras as they went about their normal daily lives. The head-mounted cameras were set to ‘time-lapse’ mode and took an image every 30 s, with images stored in RAW format (with no internal white balance correction). The color calibration of the GoPro cameras allowed for accurate estimation of the colorimetric properties of the environment from the RAW image files. Participants wore the cameras for morning, afternoon and evening sessions which lasted until the battery ran out (for approximately 2–2.5 h), generating approximately 200–300 images per session. Participants were asked to wear the cameras as they went about their daily lives, but to remove the camera if there were concerns over safety or privacy. After any duplicate images were removed there was a total of 5406 RAW image files and all were analyzed. The contents of the images included indoor settings such as home and office environments as well as outdoor environments of the urban cityscape, the South Downs national park and coastal scenes (see Fig. 3A). All participants gave informed consent to take part and ethical approval was granted by the Sciences and Technology Cross-Schools Research Ethics Committee of the University of Sussex. Data collection and analysis was performed in accordance with the relevant guidelines and regulations including the Declaration of Helsinki.

We chose the JenAesthetics dataset and the Real-World Scenes set as Jena and Brighton have similar geographical features and properties. Both locations have a similar overall size and population density, lie on approximately the same latitude (Jena 50.9° N, Brighton 50.8° N), the surrounding area of both includes hilly countryside and natural water features, and both are within the temperate broadleaf forest biome. Both locations are cities within developed, Western European countries where lifestyles and ‘visual diets’ are likely to be similar, including exposure to both natural environments and modern building materials, architecture and interiors. Therefore, the similarities of culture, lifestyles, local environment and ecology, and seasonal daylight exposure make the real-world scenes a reasonable approximation of the natural statistics of the visual diets of the participants who provided the aesthetic ratings.

### Image analysis

Analysis of the images was carried out in Matlab (Version R2023a). Images of the JenAesthetics set were rescaled such that the longest side was 800 pixels via bicubic interpolation to analyze them as they were presented in the JenAesthetics experiments^27,53^. Both the JenAesthetics images and real-world scenes were converted from RGB to LMS tristimulus values using the Stockman, MacLeod and Johnson^54^ cone fundamentals. For the JenAesthetics images, we approximated the viewing conditions under which the ratings were obtained by converting to LMS space based on RGB spectra and gamma values of a comparable display to which they were presented experimentally. The JenAesthetics images were displayed on a BenQ T221W monitor. Without available measurements of the primary spectral power distributions of the particular display used in the JenAesthetics ratings experiment we approximated the viewing conditions with a Dell model E228WFPC LCD panel with CCFL backlight which is a similar low-to-mid-range general purpose display panel similar in age and screen technology to the BenQ T221W. For the real-world scenes, we used camera-specific RGB sensitivity functions for each GoPro camera to convert from the RAW image files to LMS tristimulus values.

For image analysis of the art images and the real-world scenes, the LMS tristimulus values were transformed to a cone-opponent color space representing the pixelwise activations of two chromatic cone-opponent channels (S/(L + M) and L/(L + M)) as well as luminance (L + M) as described by the MacLeod-Boynton^14^ chromaticity diagram. To allow for comparison between the two image sets, the luminance values for the JenAesthetics images and the real-world scenes were expressed as a proportion of the maximum possible value for that image set. The maximum luminance values were calculated by converting a theoretical maximum RGB white value to luminance (L + M). When calculating the chromatic statistics, we filtered out pixels with a corresponding relative luminance value of below 0.25% of the maximum luminance, as these dark pixels are too dark to be perceptible as a color but still may skew the chromatic image statistics as they may produce strong chromatic noise.

#### Chromatic statistics

**Figure 4:**
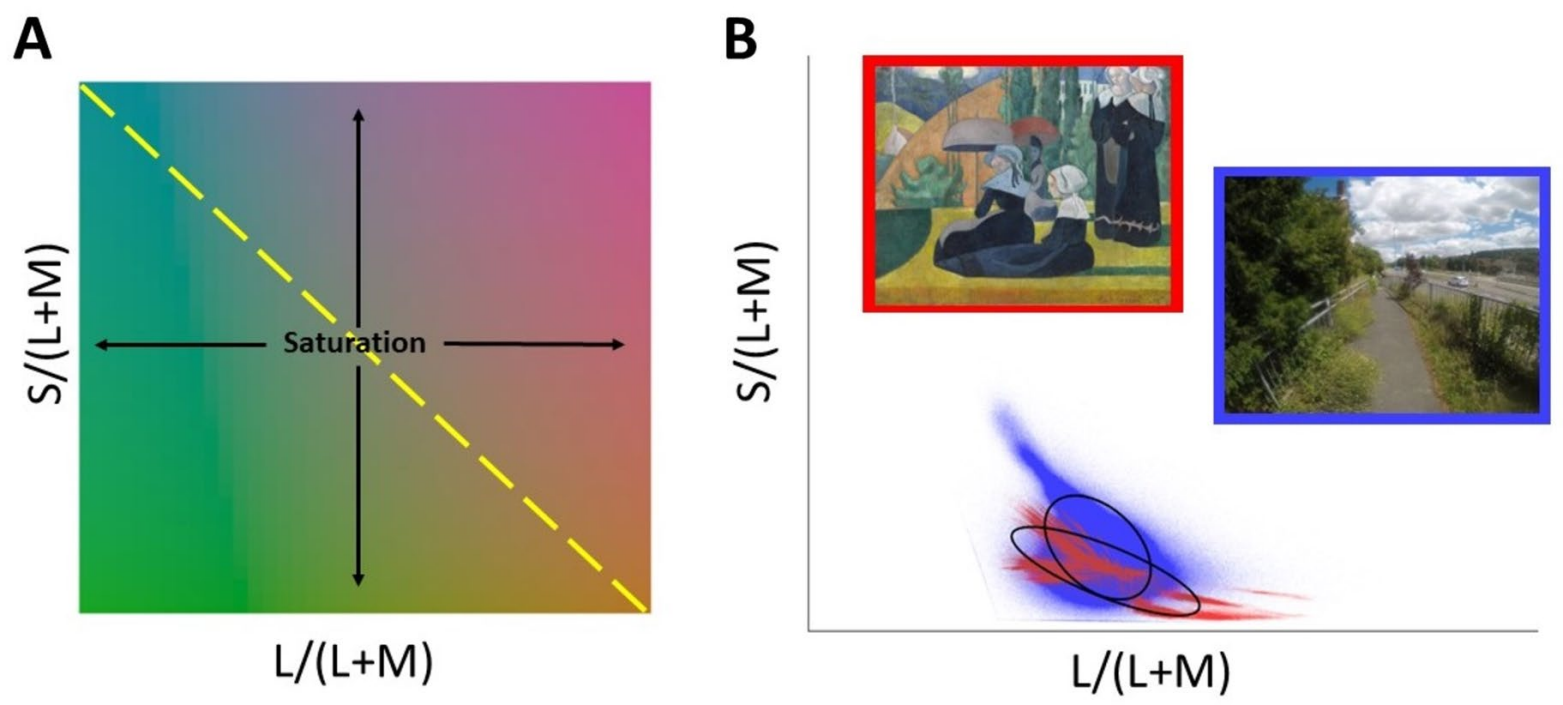
Chromatic image statistics. (**A**) the MacLeod-Boynton chromaticity diagram with the two cone-opponent axes plotted against one another and the negative diagonal that corresponds to colors that appear blue and yellow shown with the yellow dashed line. (**B**) A representative image from each of the two image sets as a thumbnail and with each pixel value plotted in the MacLeod-Boynton^14^ chromaticity diagram. A public domain image from the JenAesthetics art set is shown with a red border and plotted with red data points and an image from the real-world scenes set is shown with a blue border and plotted with blue data points. Standard deviation ellipses are shown in black fitted to each of the chromaticity distributions. The ellipse fitted to the artwork image shows lines in black indicating the ellipse axes used to calculate the ‘natural chromatic elongation’ of the chromaticity distribution.

We computed a set of chromatic image statistics using the MacLeod-Boynton chromaticity^14^ diagram (see Fig. 4A), including the mean and standard deviation of the pixel values for the two cone-opponent axes of the chromaticity diagram (S/(L + M) and L/(L + M)) and the saturation of image pixels in this chromaticity diagram. To compute saturation we converted from the Cartesian cone-opponent L/(L + M) and S/(L + M) values by subtracting the white point value from each of the values, scaling the L/(L + M) values to the same range as the S/(L + M) values, and extracting polar coordinates. We also calculated the standard deviation of the pixelwise luminance values to quantify how the luminance varied over the whole image. Images with a high standard deviation in luminance have a broader range of differences between lighter and darker pixels whereas those with a low standard deviation in luminance have a narrower range of differences between lighter and darker pixels.

We also computed two chromatic image statistics that aimed to capture the distribution of pixel chromaticities: the natural chromatic elongation and the angle of maximum color variance (as in Ref.^6^, see Fig. 4). We fit a standard deviation ellipse to the distribution of S/(L + M) and L/(L + M) chromaticities in the MacLeod-Boynton^14^ chromaticity diagram. Our ‘angle of maximum color variance’ variable (‘ellipse angle’ in Ref.^16^), was calculated as the angle of the major axis of this standard deviation ellipse and represents the chromatic axis along which the pixels of an image are biased. We then normalized the variances of S/(L + M) and L/(L + M) values in a scaled version of the MacLeod-Boynton^14^ chromaticity diagram and fitted a standard deviation ellipse to the Cartesian coordinates of pixels in this scaled chromaticity diagram. We calculated the ‘natural chromatic elongation’ (termed ‘axis ratio’ in Ref.^16^), as the log of the ratio of the ellipse axis oriented along the negative diagonal and the length of the orthogonal axis. Values greater than zero for the natural chromatic elongation describe distributions of chromaticities that are spread more widely along the negative diagonal associated with colors that appear blue and yellow, as is characteristic of natural scenes^15,16^.

#### Spatial image statistics

We computed a set of spatial image statistics that have been related to aesthetics in prior research: fractal dimension, lacunarity, spectral slope, straight and curved edge density and luminance entropy. Fractal dimension represents the extent to which self-repeating patterns across scales fill a space^10^. We measured 2-D fractal dimension which measures how a 2-D plane pattern fills a 3D space, with values ranging from 2 to 3 (as described in Ref.^55^). As the methods for calculating the fractal dimension of an image and the spectral slope (as described below) require images to be square, we cropped the images so that the longest sides were trimmed to the same length as the shorter sides for these two image statistics, keeping the largest possible central square of the image. Lacunarity characterizes how heterogeneous the spatial patterns comprising an image are and captures the rotational invariance of an image^42^. For example, images with more gaps between patterns generally have higher lacunarity. We computed lacunarity using a gliding box counting algorithm and calculated the mean lacunarity across different box sizes^56^. The spectral slope of an image describes how Fourier amplitude varies at different spatial scales. The spectral slope in natural scenes of 1/f ^*α*^ where f is the spatial frequency and *α* is approximately 1 (e.g.,^12^) represents a characteristic pattern where amplitude decreases as spatial scales become finer. Straight and curved edge density describe the proportion of pixels in an image that made up edges, separately for curved and for straight edges. We used the gradient-based connected component algorithm (adapted from the method described in Ref.^43^) to calculate this. Entropy refers to the degree of disorderliness or predictability of the information in an image. An image with a low level of entropy has a simple texture made up of highly predictable spatial patterns whereas an image with high entropy has a disordered spatial arrangement with little uniformity in composition. We calculated the Shannon entropy using the ‘entropy’ function in Matlab (R2023a).

### Analysis strategy

As in Mather’s^5^ analysis of the JenAesthetics Dataset, we used partial least squares regression (PLSR) to model the subjective ratings of beauty based on the image statistics of the artworks. Some of the image statistics used in our analyses are significantly correlated, which is incompatible with standard multiple regression analysis approaches. To allow our analyses to include variables that are correlated but that have critical differences, we used the PLSR method (as in Ref.^5^). The PLSR method overcomes the requirement for no multicollinearity among predictor variables by transforming the predictors into components (linear combinations of the original predictor variables) which can then be used in a regression to model the dependent variable^57^, in this case the beauty ratings of the artworks. To decide on the number of components to include in our PLSR models, we examined the amount of variance explained when including only the first component, then repeated this step adding in each additional component until all were included in the model. We then found the asymptote for variance explained where adding in another component did not explain more variance and chose the number of components for the final model based the lowest number of components that explained 85% of the asymptotic value for variance (after Mather^5^). To interpret the final model in all analyses, we relied both on the variance explained and we assessed the role of each predictor through their variable importance in projection (VIP) scores. The VIP score represents the contribution that a variable makes to the model and is calculated as the sum of squared correlations between the PLS components and the original variable (see “plsregress” function in MATLAB)^57^. Components with a VIP score greater than 1 are more important than average. To identify important VIPs we used a threshold VIP score of > 1.25 as this is recommended in the case of high correlations among predictors^5^. In order to assess whether the variance explained by PLSR was statistically significant, we used a permutation approach by repeating the PLSR analysis 10,000 times with random permutations of the beauty ratings for the artworks. Where the variance explained by the main analysis was greater than the 95th percentile of the variance explained in the permutation analyses, we accepted the model and proceeded with interpretation. All analyses were applied to our subset of JenAesthetics artworks across genres, as well as separately for the landscape and portrait images.

We first conducted PLSRs on the original linear versions of the image statistics of the art. However, in order to address our main hypothesis that aesthetic appreciation of art is calibrated to the image statistics of real-world scenes, we also conducted PLSRs with the image statistics of the artworks expressed relative to those of the real-world scenes. For each artwork we took the value of the image statistic calculated from the image and subtracted the mean value for the corresponding image statistic in the real-world scene set. We next divided those values by the standard deviation of the set of image statistics from real-world scenes producing a z-score value. The z-score represents the degree to which an image statistic for a given artwork deviates from the average value for that image statistic in real-world scenes, in units of standard deviation (see^58^). We analyze the absolute value of the z-score which represents the extent to which the image statistic of the artwork deviates from real-world scenes, regardless of whether it is above or below the mean.

Before entering the predictors (either as raw image statistics or expressed relative to real-world values) into PLSRs we rank-inverse normal transformed. This produces perfectly normal distributions of predictors (except in the case of tied ranks), and avoids any statistical artefacts arising from non-normal predictors. This was an important consideration, especially for image statistics expressed relative to real-world values, where the distributions of absolute z-scores could be highly skewed.

### Supplementary Information

Supplementary Information.

## Data Availability

The datasets generated during and/or analyzed during the current study are available in the OSF repository [https://osf.io/72xuq/]. We provide the values for our analysis of the raw image statistics of the JenAesthetics art stimuli, the raw image statistics of the real-world scenes, and the image statistics of the Jen Aesthetics art stimuli expressed relative to the real-world scene set. The JenAesthetics dataset is available with the permission of Christoph Redies et al. on reasonable request (see^27^). The real-world scene set is not publicly available due to containing images where participants can be identified or containing personal information such as inside people's homes and family lives. We therefore provide the raw image statistics of the stimuli rather than the stimuli and image analysis code. We make our data analysis code available, and provide the links to any open access code made available by others, in the same OSF repository.
