# Supplementary material for: The perceived beauty of art is not strongly calibrated to the statistical regularities of real-world scenes
Source: Sci Rep. 2024 Aug 21;14:19368. doi: 10.1038/s41598-024-69689-6 (PMC11339329; doi:10.1038/s41598-024-69689-6)
Supplement: Supplementary file 1 — Supplementary Information. [file 41598_2024_69689_MOESM1_ESM.docx]

**Supplementary Information:** The perceived beauty of art is not strongly calibrated to the statistical regularities of real-world scenes.

Figure S1A


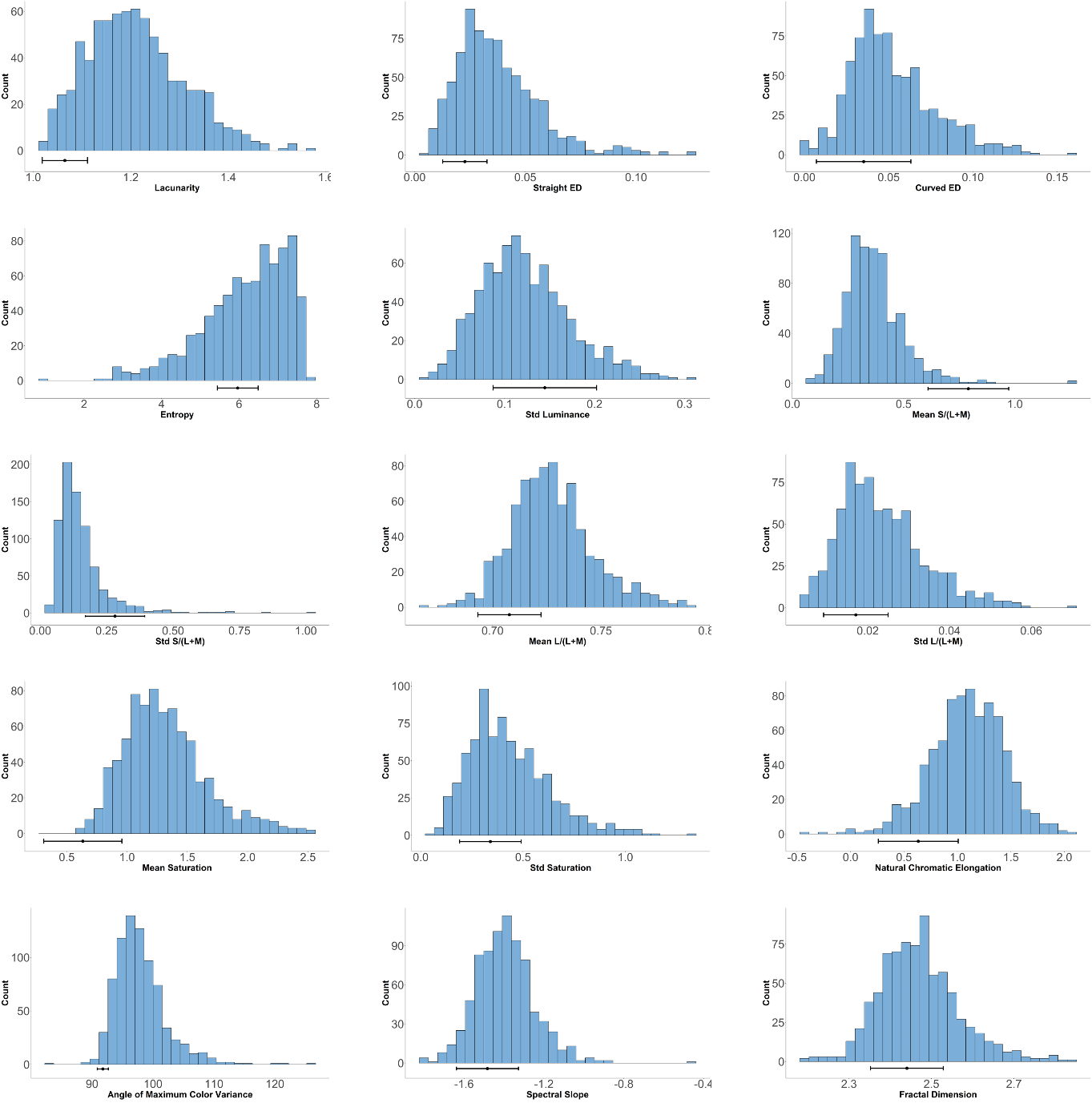


Figure S1B


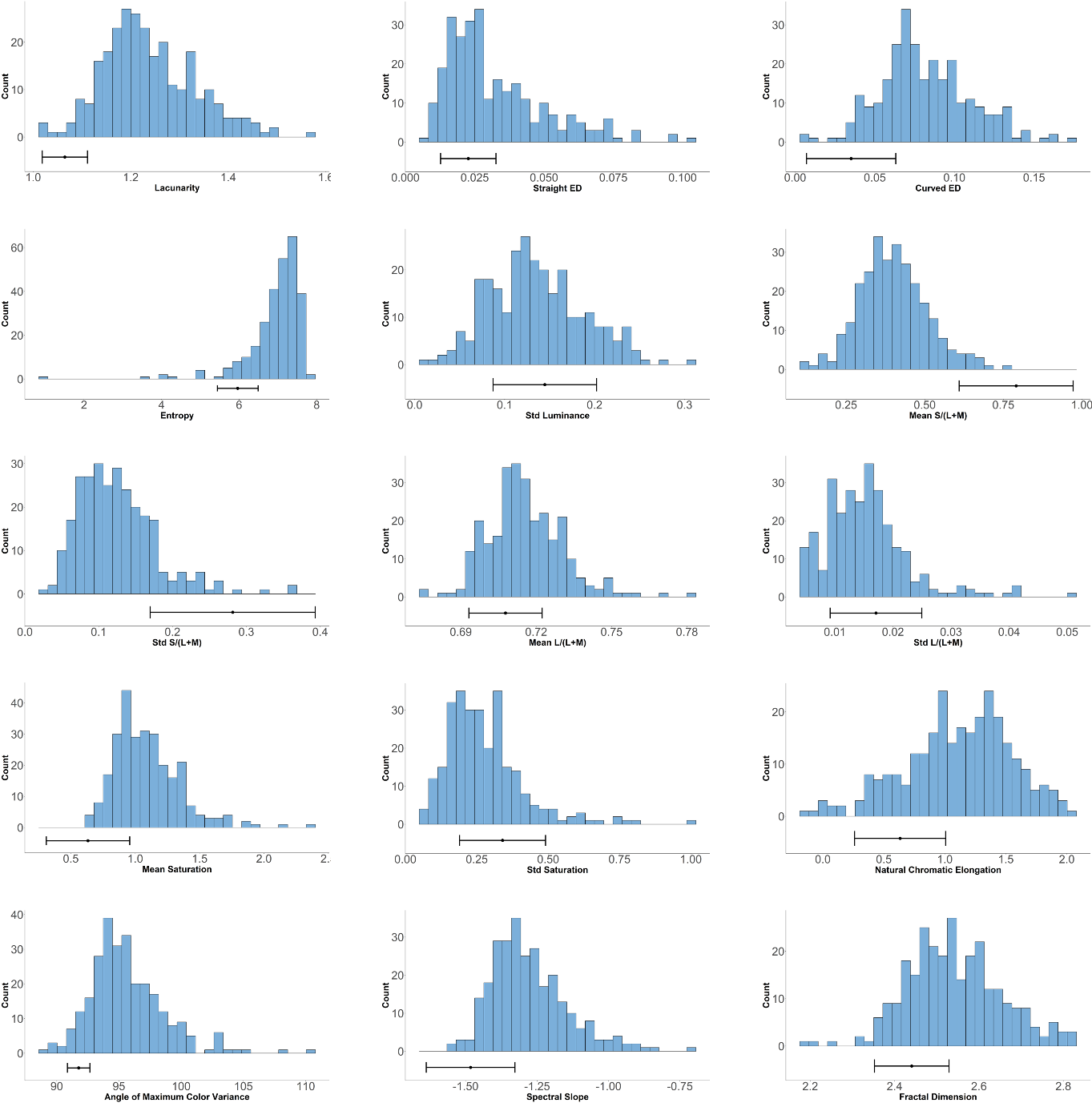


**Figure S1.** Histograms of the image statistics of the selected JenAesthetics artworks (all genres: S1A; landscapes: S1B), with the real-world scene mean (+/- 1SD) for that image statistic given below the histogram.

**Supplementary Table S1.** VIP scores from each of the PLSR models using the art’s image statistics (Art), and the art’s image statistics expressed relative to the image statistics of the real-world scenes (Real-world) to predict beauty ratings for all genres of paintings, and landscapes and portraits separately. The VIP scores for the real-world portrait PLSR are not shown since the variance explained by this model was not greater than the 95^th^ percentiles of the variance explained by the permutations. Values above the criterion of 1.25 are shown in bold.

| Image statistic | Art  all genres | Art  landscapes | Art  portraits | Real-world  all genres | Real-world  landscapes |
| --- | --- | --- | --- | --- | --- |
| Lacunarity | 0.96 | 0.69 | 1.11 | 1.09 | 0.13 |
| Straight Edge Density | 0.56 | 0.91 | **1.46** | 0.30 | **1.67** |
| Curved Edge Density | 0.70 | **1.27** | 0.88 | 1.22 | **2.03** |
| Entropy | 1.19 | 0.85 | 1.00 | 1.07 | 0.96 |
| Standard Deviation of Luminance | **2.12** | **2.61** | **2.33** | 0.23 | 0.48 |
| Mean S/(L+M) | 1.06 | 0.45 | 0.32 | **1.34** | 0.51 |
| Standard Deviation of S/(L+M) | 0.43 | 1.06 | 0.74 | 0.30 | **1.53** |
| Mean L/(L+M) | 0.79 | 0.28 | 0.29 | 0.91 | 0.14 |
| Standard Deviation of L/(L+M) | 1.07 | 0.97 | 0.50 | 0.75 | 0.77 |
| Mean Saturation | 0.81 | 0.12 | 0.35 | 1**.**10 | 0.30 |
| Standard Deviation of Saturation | 0.81 | 0.83 | 0.62 | 0.59 | 0.74 |
| Natural chromatic elongation | 0.73 | 0.62 | 0.21 | 1.15 | 1.10 |
| Angle of Maximum Color Variance | 0.91 | 0.71 | 0.46 | **1.30** | 0.69 |
| Spectral Slope | 0.93 | 0.60 | **1.56** | **1.44** | 1.06 |
| Fractal Dimension | 0.77 | 0.48 | 0.52 | 1.04 | 0.50 |
